# Evidence of Systematic Triggering at Teleseismic Distances Following Large Earthquakes

**DOI:** 10.1038/s41598-018-30019-2

**Published:** 2018-08-02

**Authors:** Robert T. O’Malley, Debashis Mondal, Chris Goldfinger, Michael J. Behrenfeld

**Affiliations:** 1 Department of Botany of Plant Pathology, Cordley Hall 2082, Oregon State University, Corvallis, OR 97331-2902 USA; 2 Department of Statistics, 239 Weniger Hall, Oregon State University, Corvallis, OR 97331-4606 USA; 3 College of Earth, Ocean, and Atmospheric Sciences, 104 CEOAS Administration Building, Oregon State University, Corvallis, OR 97331-5503 USA

## Abstract

Earthquakes are part of a cycle of tectonic stress buildup and release. As fault zones near the end of this seismic cycle, tipping points may be reached whereby triggering occurs and small forces result in cascading failures. The extent of this effect on global seismicity is currently unknown. Here we present evidence of ongoing triggering of earthquakes at remote distances following large source events. The earthquakes used in this study had magnitudes ≥M5.0 and the time period analyzed following large events spans three days. Earthquake occurrences display increases over baseline rates as a function of arc distance away from the epicenters. The *p-*values deviate from a uniform distribution, with values for collective features commonly below 0.01. An average global forcing function of increased short term seismic risk is obtained along with an upper bound response. The highest magnitude source events trigger more events, and the average global response indicates initial increased earthquake counts followed by quiescence and recovery. Higher magnitude earthquakes also appear to be triggered more often than lower magnitude events. The region with the greatest chance of induced earthquakes following all source events is on the opposite side of the earth, within 30 degrees of the antipode.

## Introduction

The number of high magnitude earthquakes has been increasing in recent years^1^, with large events clustered in time^2^, though there is considerable debate^3,4^. It is not uncommon to encounter speculation on whether a large earthquake in one part of the world has somehow managed to influence the onset of large earthquakes in other parts of the world, often days afterward. However, scientific observations of one earthquake triggering another at teleseismic distances are mostly limited to the initiation of small magnitude (≤M4) events^5,6^. Any triggering of larger magnitude events in the current catalogs is thought to be limited to those with a delay time >8 hours^7,8^. For seismologists, the probabilities of earthquake occurrences are conceptualized and derived from the historical earthquake catalog^9^. Any connections between distant earthquakes fall outside of the basic premise of the law of small numbers^10,11^. The assumption remains that there is effectively no temporal and spatial dependence beyond the aftershock region.

The current understanding on the mechanics of how one earthquake could initiate another while being widely separated in distance and time has largely been conjectural and speculative. Dynamic loading has been proposed as a mechanism for initiating remote slow-slip events, potentially impacting larger events at distance^12^. Earthquake occurrences have also been correlated with earth tides combined with ocean loading^13^. The suggestion that small perturbations from the passage of seismic waves can cause the excitation of fault systems globally has been controversial. Nevertheless, recent observations reveal interactions between distant earthquakes and fault systems. For example, the Landers (1992) and Denali (2001) earthquakes have been implicated as triggering the Yellowstone geyser system and earthquakes up to magnitude (M) 2.1, some 1,200 and 3,100 km distant, respectively^14–16^. Similarly, S-waves from the Sumatran earthquakes (2004, 2005) apparently triggered seismicity up to M = 4 in Tibet^17^, while S-waves from the Tohoku (2011) earthquake were linked to San Andreas and other fault activity across the globe^6^. The 2012 Mw8.6 earthquake in the Indian Ocean singularly in the instrumental record may have triggered several Mw >5.5 events globally^7^. A search of the catalog for rate changes and possible triggered events following 260 Mw >7 events suggested only 2–3% of them may have triggered low magnitude events within 24 hours of the mainshock^8^. Previously, Parsons and Valasco (2011)^18^ found no increase within 16 to 100 hours in M > 5 earthquakes at ranges greater than 1000 km following 205 M > 7 events. Parsons *et al*.^8^ found no systematic linkage between triggered events and mainshock magnitude, peak ground acceleration, focal mechanisms or amplitude spectra. In any case, fault activities initiated by small perturbations associated with these distant and varied sources implies that the responding locations were likely close to the end of their seismic cycles, otherwise the extremely small strains involved would be insufficient for triggering^19^.

For this study we consider the possibility that triggering of significant earthquakes (≥M5.0) occurs up to three days after the initiating high magnitude source events. The current objective is to present both quantitative and visual evidence of triggering, independent of the possible mechanics involved.

## A Test of Hypothesis

**Figure 1:**
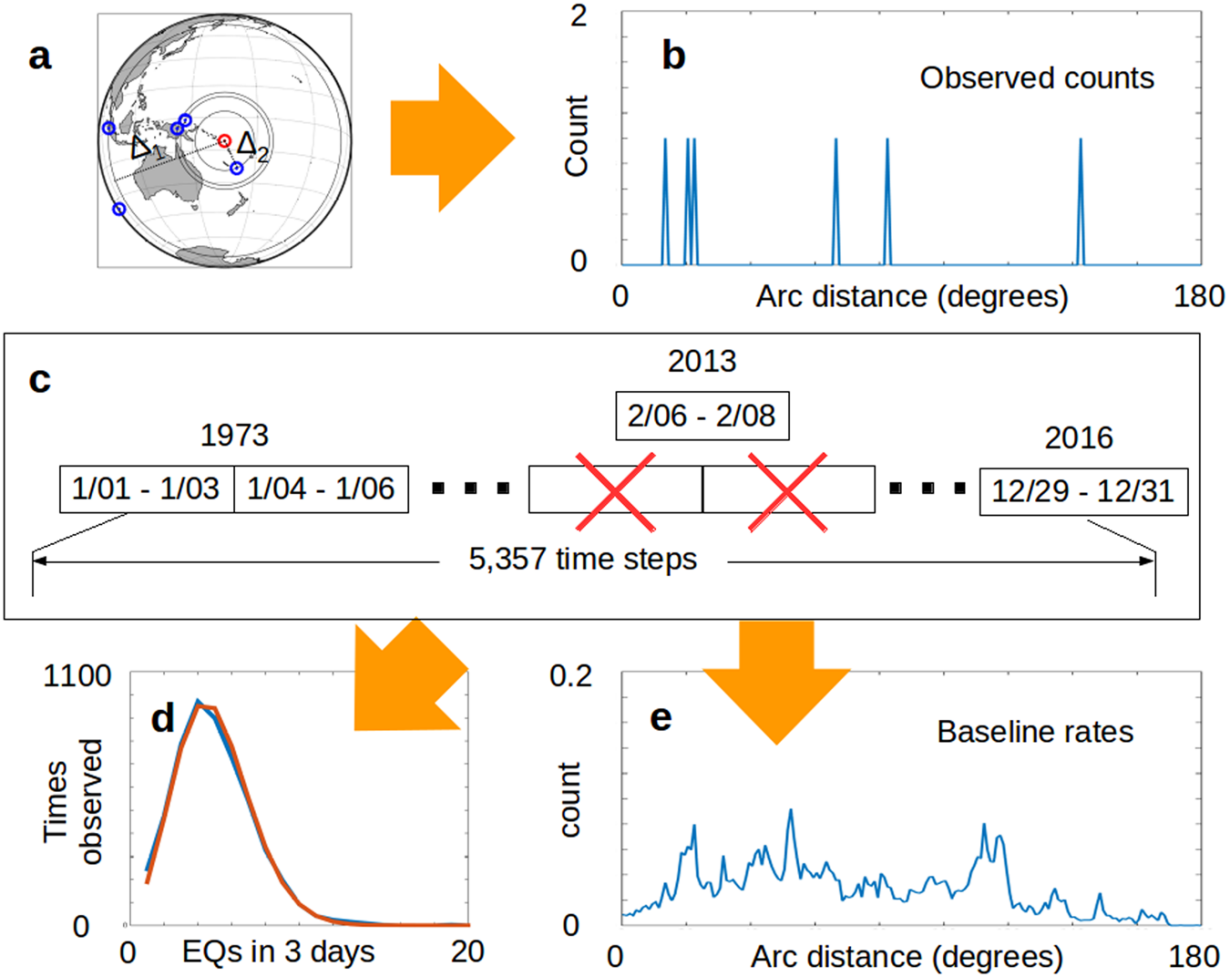
Experimental Design. (**a**) Arc-distance distribution^36^ (Δi) of earthquakes (blue circles) in the three days following the M8.0 earthquake (red circle) that took place on February 6, 2013. (**b**) The global distribution of earthquakes as a function of arc-distance for (a). (**c**) The control group extends from 1973–2016 and uses the same origin and three day window as (a,b). (**d**) Earthquake count histogram of the control group once aftershocks are removed (blue), compared to a Poisson distribution (orange). (**e**) Average three-day baseline rate of the control group for (a).

If large magnitude source events are the cause of triggering, then a measurable effect should be a systematic increase in the number of earthquakes that follow over baseline rates. We determine this by calculating how far away subsequent earthquakes are from a given source event (Fig. 1a) and then tally the observed counts over three days as a function of arc distance (Fig. 1b). This count then gets compared with the historic distribution of earthquakes^20^ as seen from the source event’s frame of reference (Fig. 1c).

A useful first step, however, is removing aftershocks from the archived data (*Methods*). This avoids anomalously high spikes in the observed counts due to known clustering processes^21,22^. The result is an earthquake count distribution that reflects the Poisson model under the basic premise that global earthquakes are independent in space and time (Fig. 1d). This becomes the null hypothesis.

We now have the setting for a test of hypothesis. Each test case (Fig. 1a,b) effectively represents a single three-day window ‘injected’ with a large magnitude earthquake suspected of inducing earthquakes, and is accompanied by a control group of 5,355 time periods that received no such injection (Fig. 1e). On the one hand, we are saying that the three-day period following a large earthquake has an increased chance of occurrence of subsequent earthquakes at certain arc distances, while the null hypothesis asserts that earthquakes have the same chance of occurring in each three-day window. This leads to a test of hypothesis that directly applies probabilities based on the binomial distribution (Methods; Supplemental Information). The smaller the resulting probabilities, the stronger the argument against the null hypothesis. Probabilities less than 5% (0.05) are commonly seen as arguments for the alternative hypothesis being tested.

## Results

### Experiment 1

**Figure 2:**
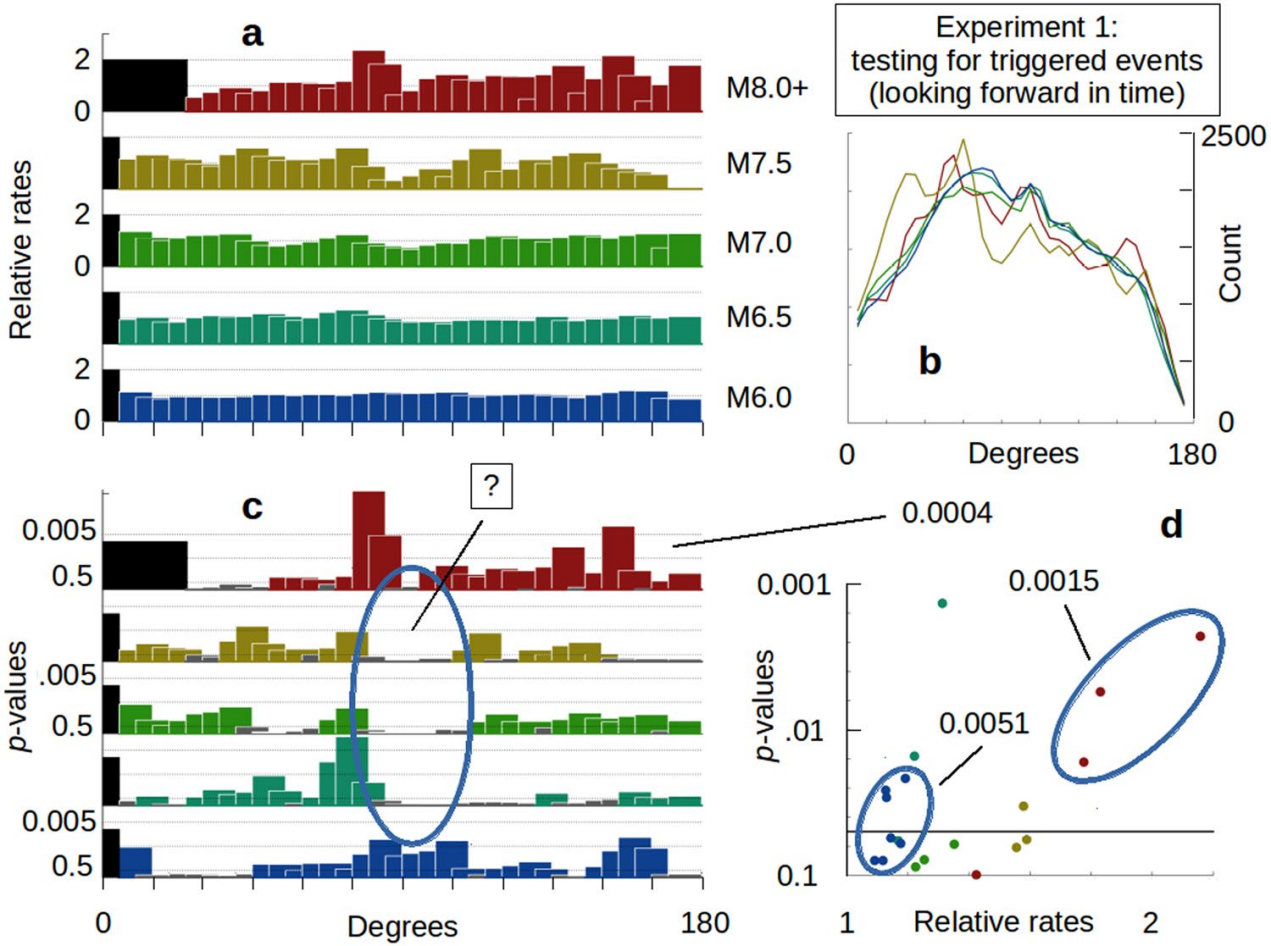
Results for Experiment 1. (**a**) Relative rates of triggered earthquake occurrences for combined source test sets: M6.0 (n = 678), M6.5 (n = 253), M7.0 (n = 101), M7.5 (n = 31), and ≥M8.0 (n = 33). Unless otherwise indicated, all magnitude ranges are narrowly defined. For example, ‘M6.0’ only includes data that are greater than or equal to M6.0 but less than M6.1. 0x, 1x and 2x over baseline shown. Relative rates = observed test-set rates divided by longterm baseline rates. Bins are ten degree averages with five degree offsets. The first 25 degrees are omitted from M8.0+ to avoid potential leakage of unfiltered aftershocks; the remainder omit the first 5 degrees. (**b**) The distribution of the average control group population is shown for each of the test sets shown in (a). (**c**) Statistical *p-*values for the relative rates given in (a), plotted as [-log10(*p-*value)]. *P*-values less than 0.5 are shown in grey. A zone of unusual quiescence is indicated from 80 to 100 degrees for M6.5 and greater. (**d**) Summary of the relative rates and *p*-value results from (a,c), for individual ten degree bins with *p*-values < 0.1. *P*-values for groups of data from a given test set are shown, including the *p*-values for the M8.0+ set for bins from 100 to 175 degrees (see text).

We investigated earthquake triggering using two complementary numerical experiments. In *Experiment 1* we pursue a meta-analysis^23^ of all events in a given test-set. Effectively, we look for what is common when the results of the test-set are combined, rather than seeking isolated patterns based on individual earthquakes. Test-sets are compiled of potential sources (*Methods*) and we look forward in time to see if there are any systematic increases in the distribution of subsequent earthquakes indicative of triggered events (Fig. 2a). We analyze these in ten degree bins, offset every five degrees. The results are visualized by plotting how many times the observed test-set rates are over baseline levels (i.e., relative rates). We will not use data that are in the aftershock zone, which we define as 3 times the rupture length of the fault. For the largest magnitude events, that extends just over 25 degrees and the first available bin will be centered at 30 degrees. For M7.5 events and less we will simply omit the first bin.

The sensitivity of triggering to source event magnitude is probed by using test-sets based on magnitude ranges that are narrowly defined. For example, ‘M7.0’ only includes data that are greater than or equal to M7.0 but less than M7.1. We find that large source events typically yield relative rates between 1x and 2x over baseline when examined in ten degree bins (Fig. 2a). The average number of observations per source event in our control group (Fig. 2b) is shown in case there are concerns that any large relative rates are due to insufficient numbers of control group observations. Specific 10-degree bins show *p-*values that are less than 0.05 (Fig. 2c). What is interesting, however, is when the *p*-values are considered in combination (Fig. 2d). For example, the chances of a uniform distribution having at least three out of 30 M8.0+ samples with *p*-values of 0.0167 (or less) is 0.0015. Extremely small individual *p*-values like the M8.0+ data aren’t required for this type of result: the chances of a random distribution having at least seven out of 35 M6.0 *p*-values of at least 0.0789 is 0.0051. If we just consider the possibility of a random distribution of having 13 of 16 estimates with p-values of no more than 0.4401 (as seen for the M8.0 data from 100 to 175 degrees), we get 0.0004. Such examples of low collective *p-*values (0.0015, 0.0051 and 0.0004) all suggest the premise of triggering.

While relative rates >1 are of interest as a possible indicator of triggering, relative rates <1 can also be interesting, especially if they are systematic. There is a stretch of very curious low relative rates associated with M6.5 and greater from 80–100 degrees away from the source events (Fig. 2c). The chances of that being a random expression has a p-value of 0.0013, and is in contrast to the strong response of the M6.0 data. Such an expression of systematic relative rates lower than 1.0 can be an indicator of a zone of quiescence or suppression following large events. We will see this expressed again in subsequent results.

### Experiment 2

**Figure 3:**
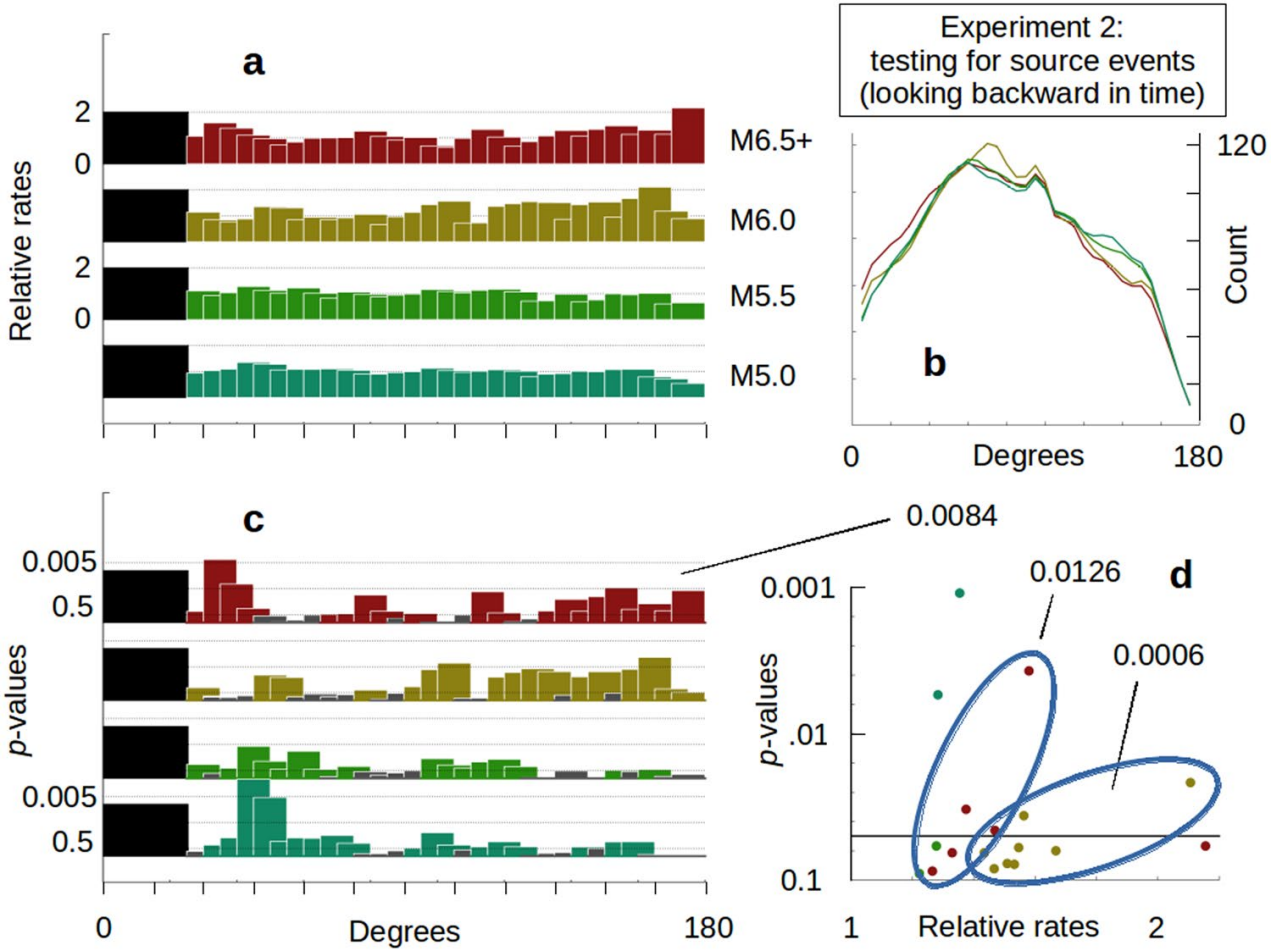
Results for Experiment 2. (**a**) Relative rates of triggered earthquake occurrences for combined source test sets: M5.0 (n = 4,409), M5.5 (n = 1,769), M6.0 (n = 678), and ≥M6.5 (n = 1,417). Unless otherwise indicated, all magnitude ranges are narrowly defined. For example, ‘M6.0’ only includes data that are greater than or equal to M6.0 but less than M6.1. 0x, 1x and 2x over baseline shown. Relative rates = observed test-set rates divided by longterm baseline rates. Bins are ten degree averages with five degree offsets. The first 25 degrees are omitted to avoid potential leakage of unfiltered aftershocks. (**b**) The distribution of the average control group population is shown for each of the test sets shown in (a). (**c**) Statistical *p-*values for the relative rates given in (a), plotted as [-log10(*p-*value)]. *P*-values less than 0.5 are shown in grey. (**d**) Summary of the relative rates and *p*-value results from (a,c), for individual ten degree bins with *p*-values < 0.1. *P*-values for groups of data from a given test set are shown, including the *p*-values for the M6.5+ set for bins from 100 to 175 degrees (see text).

In *Experiment 2* we explore triggering based on the magnitude of the induced events. Test-sets are now compiled of potentially triggered events (*Methods*) and we look backward in time for the distribution of available source events (earthquakes ≥M6.5). Spatial distributions of relative rates (Fig. 3a), average control counts (Fig. 3b) and *p*-values (Fig. 3c) are shown as before. The first available bin is again centered at 30 degrees to avoid potential contamination of results from the traditional aftershock zone from the largest magnitude earthquakes. If we examine the *p*-values of individual bins for a given class of data (Fig. 3d) we again see very small *p*-values resulting (0.0006 for the eight M6.0 values shown in Fig. 3d, and 0.0126 for the six M6.5+ data). The data in the opposite hemisphere for the highest magnitude set again shows very low probability of random occurrence (*p*-value = 0.0084). The examples of low collective *p-*values (0.0006, 0.0126 and 0.0084) from this experiment again support the premise of triggering.

### Global Patterns

The patterns seen in the relative rates between test-sets have similarities (Figs 2a and 3a), but may express the results of under-sampled distributions. We therefore ran additional test-sets through *Experiment 2* to better define the distribution of possible source events. In order to compare the relative rates between test sets that have different sample sizes, we normalized all *Experiment 2* results to 1200 samples (*Methods*). Our aim is to see, first, what the average pattern of relative rate response for the distribution of source events looks like, and second to define a pattern for the upper bound of the response.

**Figure 4:**
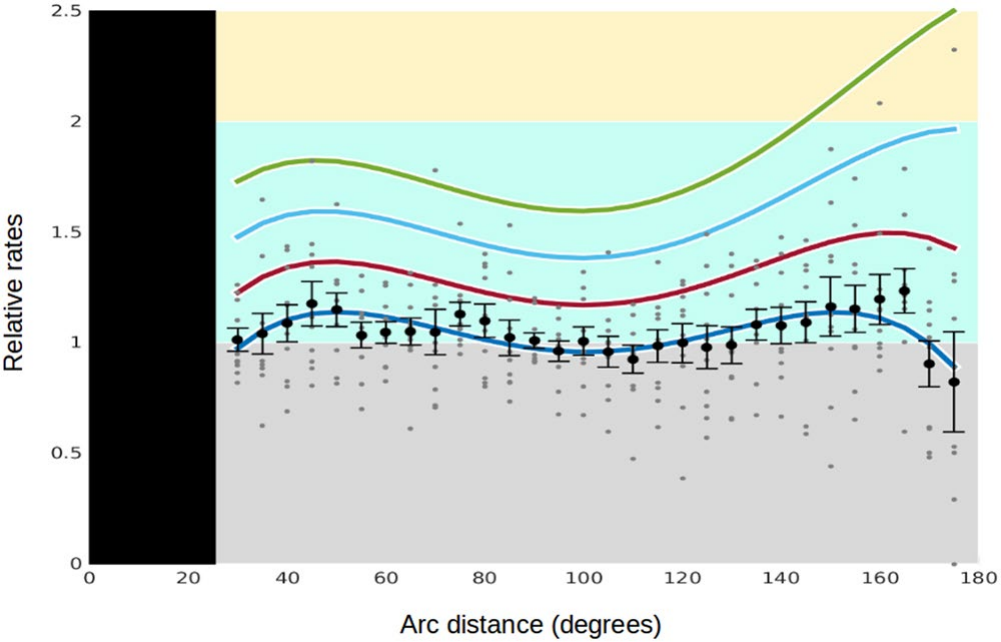
Collective Results from Experiment 2: Global Average Response and Upper Bound. (**4**) Summary plot of additional test sets run through *Experiment* 2: M5.0 (n = 4,409), M5.5 (n = 1,769), M5.6 (n = 1,520), M5.7 (n = 222), M5.8 (n = 919), M5.9 (n = 856), M6.0 (n = 678), M6.1 (n = 551), M6.2-M6.4 (n = 1,116), and ≥6.5 (n = 1,417). Relative rates were normalized to a sample size of n = 1,200 for all sets (*Methods*). Unless otherwise indicated, all magnitude ranges are narrowly defined. For example, ‘M6.0’ only includes data that are greater than or equal to M6.0 but less than M6.1. Average relative rates for each ten-degree bin are plotted in black along with their standard error to indicate how much that could vary. Relative rate data for each test set are plotted in grey. A 4^th^ order polynomial fit to the average observed relative rate for each bin is shown as the dark blue curve, as well as curves for 1, 2 and 3 standard deviations above the average. An appropriate upper bound for the maximum relative response appears to be between 2 and 3 standard deviations above the average.

The average relative rate for each of our ten degree bins is shown along with the standard errors (Fig. 4). Standard errors help assess the possible range of the average values. A 4^th^ order polynomial is fit to the averages (dark blue curve, Fig. 4), along with curves for one, two, and three standard deviations away. If we consider a representation of the upper bound to be somewhere between 2 and 3 standard deviations away from the average, then it appears that the potential maximum response from a source event expresses itself in the antipodal region, starting at perhaps 140 to 150 degrees away from the source and exceeding relative rates of 2.0 for 10-degree bins. The average response appears to have maxima near 45 degrees and 150 degrees, with minimal impact at 90 degrees. Expressions of potentially coherent spatial patterns as seen in the highs and lows of the average trace, or the asymmetry of the upper bound, are again in contrast to the random, uniform distribution of the null hypotheses.

### Relative rate as a function of magnitude

**Figure 5:**
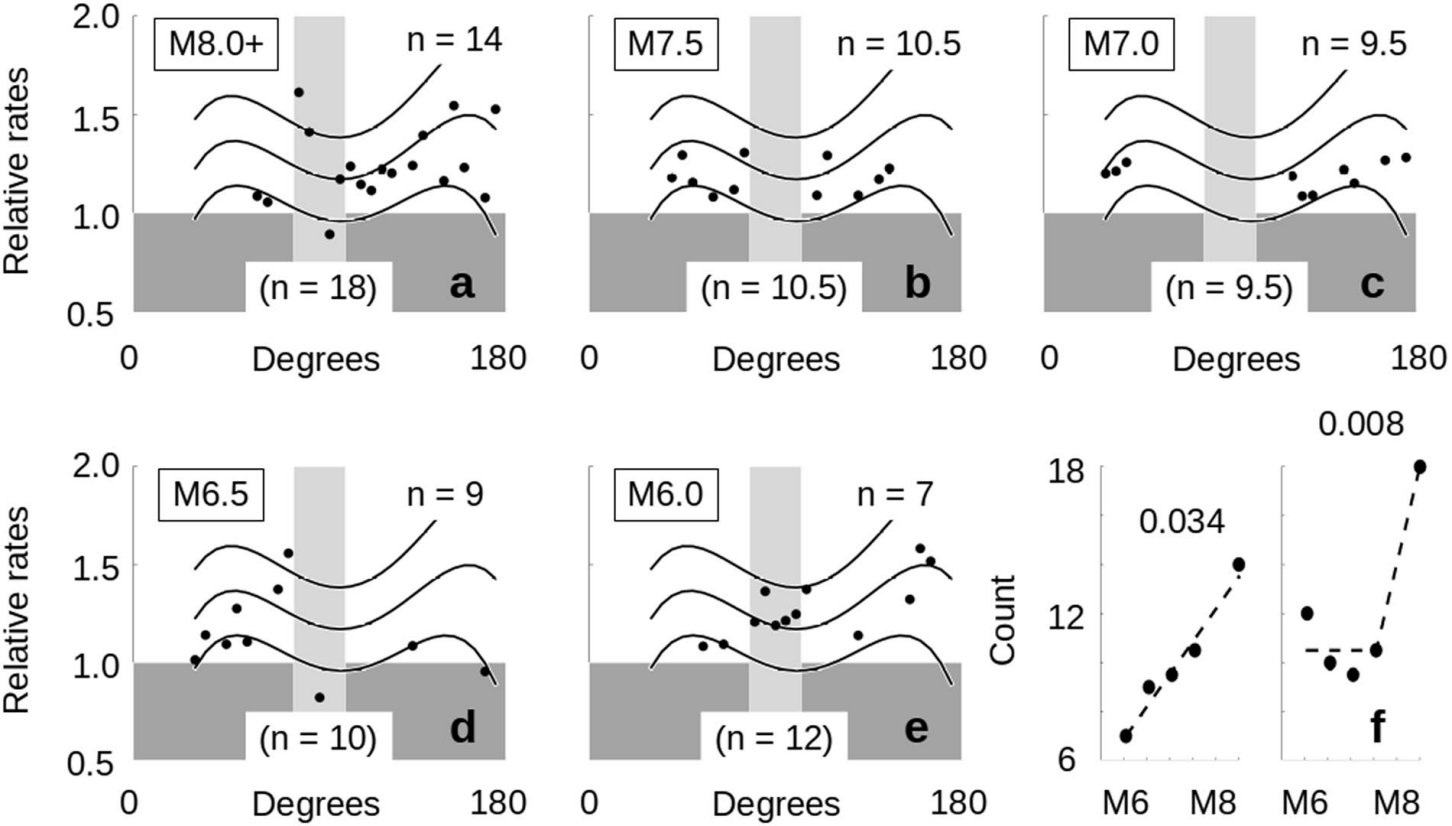
Response Rate Dependence on Source Event Magnitude. (**a**–**e**) The relative rates of the Experiment 1 data (Fig. 2a) were compared with each other after first being normalized to a sample size of 100 (*Methods*). Each ten-degree bin centered at 30 degrees to 175 degrees was examined to see how often a given test set had either the largest or the second largest relative rates between the five test sets. The data for these 1^st^ and 2^nd^ place results are shown in (a–e) for each test set. The darker grey area indicates relative rates less than 1, while the light grey area indicates the potentially quiescent zone from 80 to 100 degrees suggested in Fig. 2c. The average trace from Fig. 4 is shown, along with the 1^st^ and 2^nd^ standard deviations, as a visual frame of reference. Numbers in the upper right corner of each plot indicate the tally if the light grey zone is omitted; numbers at the base of the grey zone indicate the tally when the entire range from 30 to 175 degrees is counted. (**f**) left – summary plot for the counts as a function of magnitude if the zone from 80 to 100 is omitted. The *p*-value for the M8.0+ total (0.034) is a result of considering the number of successes vs the number of trials when the probability of success is 0.4. (**f**) right – summary plot for the counts as a function of magnitude for the entire zone from 30 to 175 degrees. The *p*-value for M8.0+ is again given for this case. (**f**) (left) indicates a linear dependence of higher relative rate values to magnitude, while (**f**) (right) only shows a dependence to be the case for M8.0+ results. Both indicate a dependence at the highest magnitudes.

There are two questions which consider potential connections between relative rates and magnitude. The first is whether source events trigger earthquakes more frequently or not as a function of magnitude, and the second is whether it is easier to trigger larger or smaller magnitude events. The first question is addressed by the results of *Experiment 1*. Because the test sets range in size, we normalize the results to a sample size of 100 (*Methods*). We then compare the adjusted relative rates for each of our five classes of data (M8.0+, M7.5, M7.0, M6.5 and M6.0) and ask how often did they have the highest or second highest relative rate for each ten degree bin (Fig. 5a–e). The result we get depends on whether or not we use the data in the potential quiescent zone for high magnitude events (shown as the light grey band). The total number of 1^st^ or 2^nd^ finishes, excluding the grey zone, is given in the upper right corner of the individual plots. The total number including the grey zone is shown at the bottom of each plot. These counts are summarized in Fig. 5f. If we take the total range of data with no exclusion zone (Fig. 5f, right), only the M8.0+ set separates itself from the results of the other magnitude data. The likelihood of a random distribution of the M8.0+ data coming in 1^st^ or 2^nd^ in 18 out of 30 heats with a probability of success of 0.40 has a *p*-value of 0.0083. If, however, we suspect the data between 80 and 100 degrees to be expressing some other process and omit that region, then we monotonically go from 14 successes for M8.0+ down to 7 successes for M6.0 (Fig. 5f, left). The *p*-value of the M8.0+ successes in this case is 0.034, but the greater significance here is that any such monotonic distribution is contrary to the null hypothesis.

**Figure 6:**
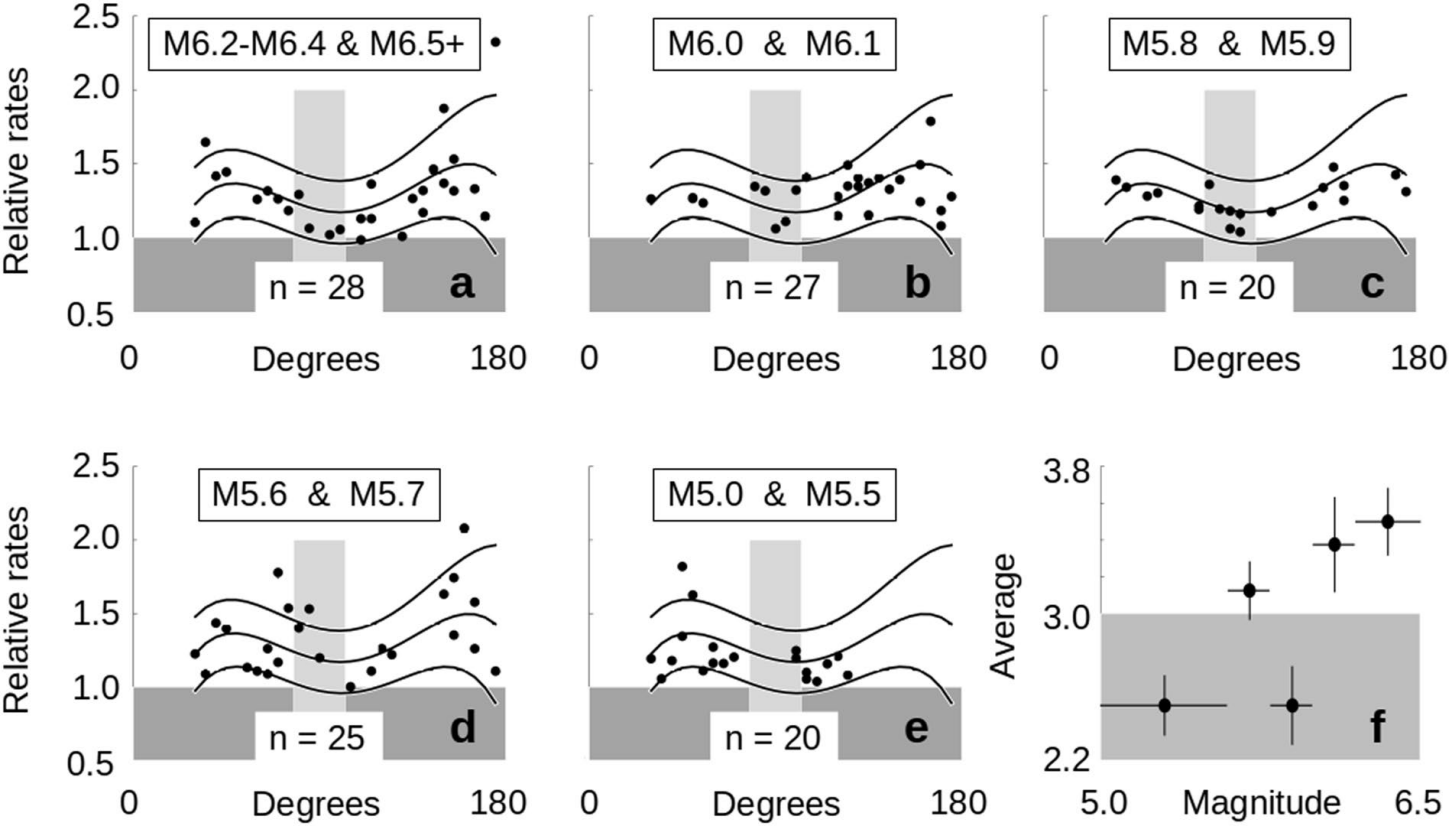
Response Rate Dependence on Triggered Event Magnitude. (**a**–**e**) The relative rates of the *Experiment 2* data used in Fig. 4 were compared with each other. These data were already normalized to a sample size of 1,200 (*Methods*). Each ten-degree bin from 30 degree to 175 degrees was examined to see how often a given test set had the largest through the fourth largest relative rates between the ten test sets. Results of adjoining magnitudes were then combined, and the data for these 1^st^ through 4^th^ place results are shown in (a–e). The darker grey area indicates relative rates less than 1, while the light grey area indicates the potentially quiescent zone from 80 to 100 degrees suggested in Fig. 2c. The average trace from Fig. 4 is shown, along with the 1^st^ and 2^nd^ standard deviations, as a visual frame of reference. Numbers at the base of the grey zone indicate the tally of combined 1^st^ through 4^th^ place finishes when the entire range from 30 to 175 degrees is counted. (**f**) We calculate the average number of times each combined set finished 1^st^, 2^nd^, 3^rd^ or 4^th^ along with the associated standard error. Black circles show the average values and the vertical black lines indicate +/− one standard error. The horizontal black lines show the min/max range of magnitudes used in the combined test sets. The grey band indicates the area that is below the expected average of 3.0. The lowest magnitude group is below the average by ~3 standard errors while the highest is similarly above it. The implication is that higher magnitude earthquakes are triggered more often than lower magnitude events.

A similar approach is taken for the data from *Experiment 2*, where we now count the top 4 finishes in each bin (keeping the random odds of success at 40%) (Fig. 6a–e). We combined the results of adjoining magnitude test runs (for example, putting the results for M6.0 and M6.1 together) and calculated the average number of times that each group finished 1^st^, 2^nd^, 3^rd^ or 4^th^, along with the standard error for that average (Fig. 6f). Surprisingly, there appears to be an increasing trend as the magnitude of the triggered event increases. The average value for the highest magnitude triggered events rises above the expected average of 3.0, while the average value for the lowest magnitude triggered events is below 3.0 by at least three standard errors. This again is in contrast to the results expected from a uniform random distribution.

### Temporal Patterns

**Figure 7:**
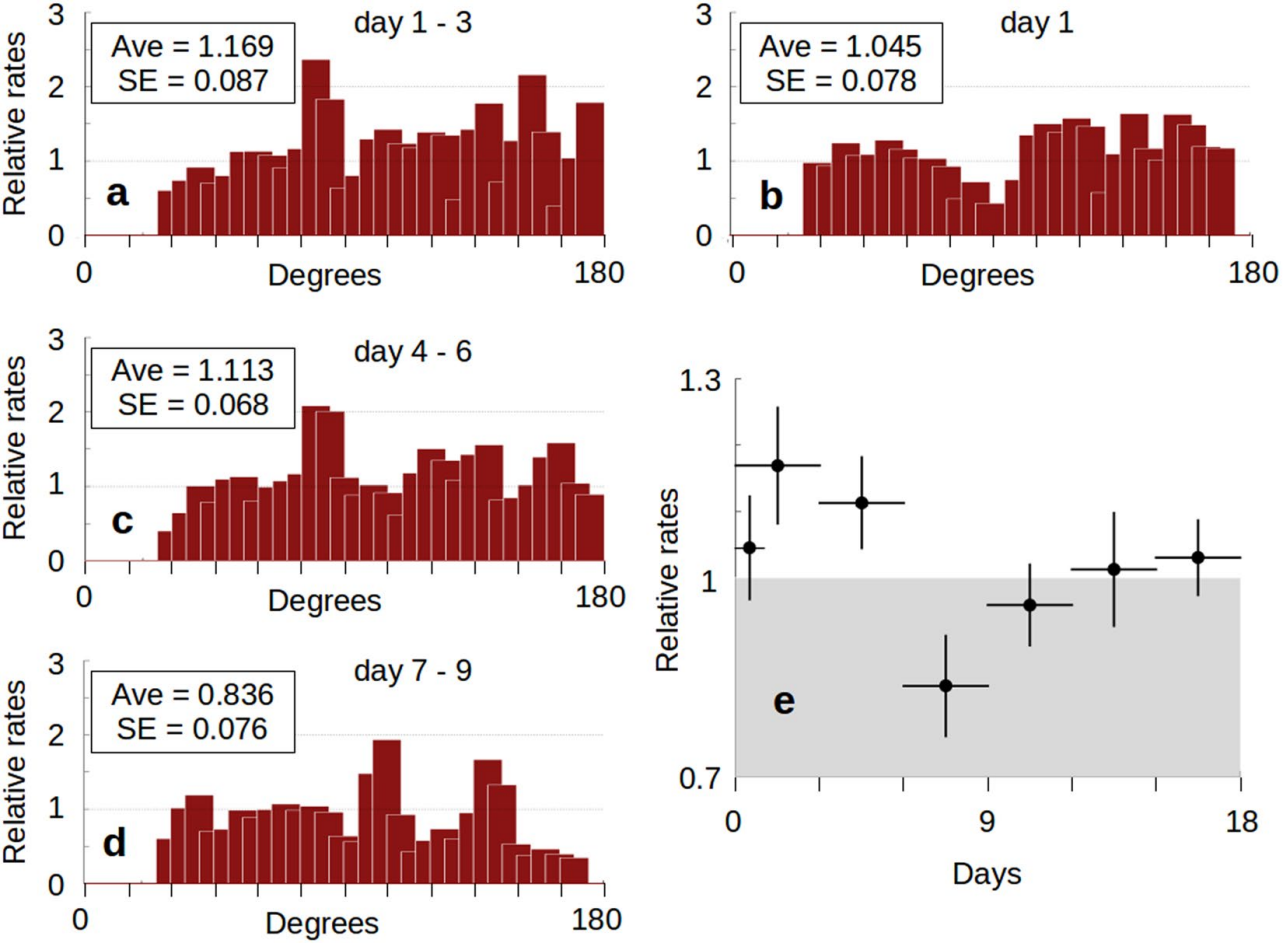
Global Relative Rates as a Function of Time. (**a**) Experiment 1 results for a three-day window for M8.0+ source events (Fig. 2a). The global average relative rates for ten degree bins from 30 to 175 degrees is indicated, along with its standard error. (**b**) Results for a one-day window following the M8.0+ source events. Relative rates are adjusted to match a three-day window (*Methods*). The global average relative rate and standard error are indicated. The quiescent zone suggested in Fig. 2c is visible in this set. (**c**) Results for a three day window with a three day lag following the M8.0+ source events. Average and standard error calculated as before. (**d**) Results for a three day window with a six day lag following the M8.0+ source events. Average and standard error calculated as before. (**e**) Summary plot of the global average and standard error of three day windows that sample 18 days total. Black circles represent the average global relative rates and the vertical black lines indicate +/− one standard error. The horizontal black lines show the coverage of the three day windows. The light grey area indicates relative rates less than 1. There appears to be an increase in the relative rates after the 1^st^ 24 hours following M8.0+ source events, which then goes into a quiescent period with lower relative rates than normal, followed by a recovery period.

We now wish to explore the change in the global average relative rate with increasing lag (Fig. 7). Figure 7a reproduces the results for Experiment 1 for the M8.0+ set of source data (Fig. 2a) and now includes the global average relative rate for ten-degree bins along with its standard error. By shifting the three-day window away from the onset time, we are able to determine the relative rates of day 4–6 (Fig. 7c) and day 7–9 (Fig. 7d). A summary of the averages and standard errors for three-day windows that extend out to day 15–18 shows an apparent period of quiescence and recharge following the initially elevated relative rates indicative of triggering.

For comparison, we also show the relative rates obtained over the first 24 hours for this test set, scaled to an equivalent three-day window (Fig. 7b). This test was initially done for comparison with previous studies that tested data from the first 24 hours. However, the spatial pattern shows a clear suppression of relative rates near the 90 degree mark, that was suggested in the previous *Experiment 1* data. Systematic patterns of relative rates, either spatial as in Fig. 7b, or temporal as in Fig. 7e, are contrary to what would be expected from the uniform, random distribution of the null hypothesis.

## Discussion

Large earthquakes appear to be associated with an increase in the number of subsequent earthquakes up to three days later in time. *P*-values taken in combination of various observed features were often less than 0.01 (Figs 2d and 3d). An upper bound of a global forcing function is calculated (Fig. 4) which has its largest impact within approximately 30 degrees of the antipode, with a minimum near 90 degrees. The largest magnitude source events (M8.0+) has the greatest number of high relative rates (Fig. 5f) with an associated *p*-value of again <0.01. It also appears that larger magnitude events are more likely to be triggered than smaller ones (Fig. 6f). Lastly, we see that global average relative rates appear to progress from a triggering onset to one of quiescence, followed by a recovery period (Fig. 7e). These observations of connected spatial, temporal, and magnitude patterns taken together are a consistent argument against the null hypothesis. While not statistical proof, they do express visual evidence of both systematic triggering and possible quiescence following large source events.

**Figure 8:**
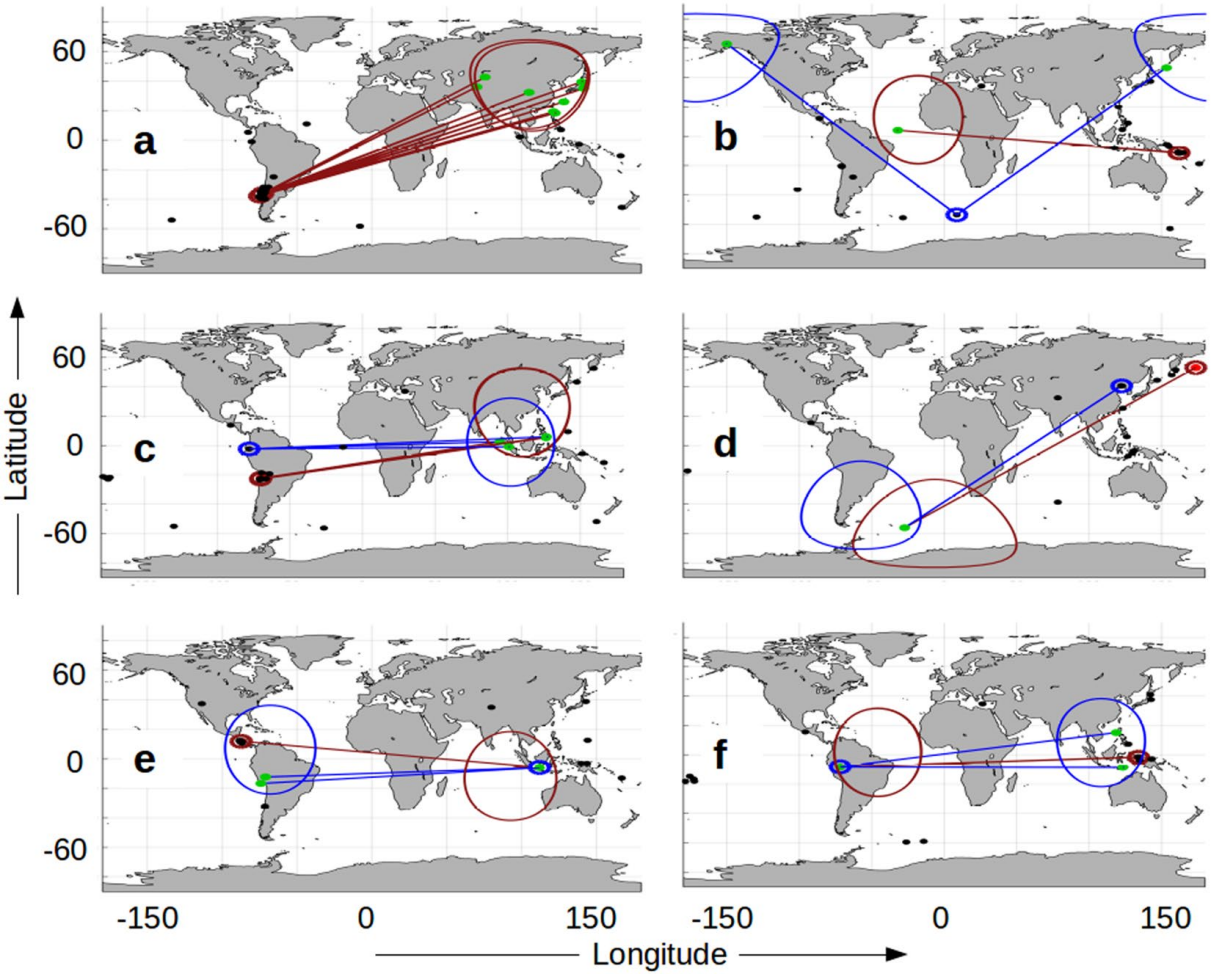
Maximal Impact Areas Following Large Source Events. Maps^36^ of earthquakes^20^ ≥M5.0 following large source events. The first event has a small red circle, and the highest global impact is outlined as a cap in the antipodal area. Earthquakes in the high impact areas within three days of the source event are in green, and connect to their source event. Second source events within three days of the first event are noted in blue. Black circles indicate earthquakes outside of the 30 degree caps and/or the three-day windows. (**a**) Single large (M8.8) Chilean event on February 27, 2010. There are ten events inside the cap area. Second, slightly displaced cap is due to large aftershocks. 3.5 days of data are shown. (**b**) The first event is M7.6 in the Solomon Islands (4/12/2014); the second event is M6.8. The areas of highest influence are globally separated over a period of 5.5 days. (**c**,**d**) When the high influence areas of two source events overlap, the potentially induced events are often in the intersection of the two caps. M7.7 (11/14/2007) and M6.8 shown in (c); M7.6 (2/2/1975) and M7.0 shown in (d). 4.5 days of data shown in both. (**e**,**f**) When the first source event initiates a second, large event, earthquake chaining follows. (**e**) illustrates M7.6 in Nicaragua (9/2/1992) followed by M6.7 in Indonesia (3.5 days of data shown). (**f**) initiates with an M7.7 event (10/10/2002) followed by an M6.9 in Brazil (5.5 days of data shown).

Examples of earthquakes that fall within 30 degrees of the antipode following large events are easy to find (Fig. 8). The outlined circular areas in the map views represents the antipodal region with the greatest chance of induced earthquakes following large magnitude source events. The upper bounds of rates of short term, increased risk for all source events, for example, are 2 times greater (or more) within that zone than typical seismicity. The largest magnitude earthquakes can trigger multiple events inside that zone, as seen following the February 27, 2010 M8.8 subduction thrust earthquake in S. Chile^24^ (Fig. 8a). It is also possible to have two source events active within the same three-day period. They can impact different regions of the globe, as seen after the April 12, 2014 M7.6 transform earthquake near the Solomon Islands, and the M6.8 earthquake occurring 55.7 hours later in the Bouvet Island region of the South Atlantic/Southern Ocean (Fig. 8b). When the antipodal areas of two source events overlap, subsequent events often appear to be within the intersection of the two zones. We see this after the November 14, 2007 M7.7 subduction thrust earthquake near Tocopilla, Chile^25^, and the M6.8 earthquake from the Peru-Ecuador border region 35.5 hours later (Fig. 8c). This is also seen following the M7.6 Near Island earthquake in the Aleutians (February 2, 1975) and the M7.0 Haicheng, China left-lateral slip earthquake 50.9 hours later^26^ (Fig. 8d). If a second source event is initiated by the first source event, we have the setting for a connected chain of earthquakes. An example of this possibility can be seen after the M7.7 subduction earthquake near the coast of Nicaragua^27^ (September 02, 1992) and the subsequent M6.7 earthquake in Indonesia that occurred 5.6 hours later (Fig. 8e). Similarly, an M7.7 shallow strike-slip earthquake near the coast of Papua, Indonesia^28^ (October 10, 2002) is followed by an M6.9 earthquake in northwestern Brazil 57.3 hours later (Fig. 8f) that connects back to additional earthquakes within its antipodal zone.

Our observations of triggering contrast with an earlier study testing on a 16-hour to 100-hour window that reports no evidence of significant triggering^18^. It may be that the methods used in this study are simply more sensitive to such small changes in relative rates. Both studies may be in agreement that within 24 hours there is minimal systematic triggering (as suggested in Fig. 7b), with triggering subsequently detected by this study beyond that time period (Fig. 7e). The previous study looked forward in time similar to our prospective analysis of *Experiment 1*, but there is clear use of different temporal binning (24 vs. 72 hours). Subtle differences exist regarding the magnitudes of the source test sets, where the previous work has grouped together what this paper has retained as separate cases. This potentially expresses what is known in probability and statistics as Simpson’s paradox^29^, where trends are present in subsets of data, but are not found when the subsets are combined. There is also a difference in the magnitude range of the possible triggered events, which the previous work limited to <M7. Our use of the retrospective analysis of *Experiment 2* suggests that higher magnitudes are more readily triggered than small (Fig. 6f) which may also be impacting the results. We also note that the papers differed in their handling of global aftershock data, which impacts baseline calculations. Removing the aftershocks was important in our case, leaving the data in a Poisson distribution (Fig. 1d) and available for direct probabilistic analysis.

In this paper, we were able to bring forward previously unseen patterns of increased global seismicity over three days following large magnitude earthquakes. Slow triggering due to cascades of small events dynamically triggered, slow aseismic deformation, or fluid migration represent potential mechanisms that could be set in motion and take days, months, or longer to complete, and could contribute to a multi-mechanism triggering. However, regardless of mechanism, the results shown here represent the first discernible evidence that large earthquakes are associated with an increase in significant events around the globe and up to three days later in time, and can potentially be used to derive improved short term earthquake forecasts and risk assessment.

## Methods

### Data Selection

Earthquake data were obtained from the United States Geological Survey (USGS) archives^20^ for all earthquakes with magnitudes ≥M5.0. The time period covered goes from the start of 1973 through the end of 2016. A minimum threshold of M5.0 for data going back to 1979 was determined to be globally detectable in a previous study^1^. This is confirmed in the current set by examining the magnitude-frequency distribution of the data. No roll off or corner frequency is observed in the data used for this study as the magnitudes drop to M5.0. Additionally, predicted *b*-value counts are within 0% to −6% of the observed totals for magnitudes below M5.5. With no observable drop-off in the earthquake counts at the lowest magnitudes used in this study, we conclude that the global catalog is complete when using a threshold of M5.0.

After filtering out aftershocks (described below) these data were used for constructing test-sets and defining the baseline rates of seismicity in our two numerical experiments.

### *Experiment 1*: A prospective analysis testing for triggered events

*Experiment 1*, looking forward in time, tests to see if large events were associated with the occurrence of subsequent earthquakes. Test-sets are created of various, large magnitude potential source events (M6.0 through ≥M8.0), and the next three days in time are examined to observe the effects of triggering on the distribution of the subsequent earthquakes ≥M5.0.

### Observed Counts

Test-sets were created based on specific magnitudes (eg: M7.5) and represent possible source events. The test set for M7.5 is narrowly defined, and only includes data that are greater than or equal to M7.5 but less than M7.6. Each earthquake in a test-set has an associated latitude, longitude and time. In *Experiment 1*, the time of each event represents the start time for the three day observation period looking forward in time. For each earthquake in a test-set, the archived data were searched for any event ≥M5.0 that took place within the next three days. The latitude and longitude of the test event represents the local origin, and the arc distance is computed from the origin to each of the earthquakes found in the three days following the test event (Fig. 1a). These data were initially spatially binned at one degree intervals, and form the basis of the observed counts (Fig. 1b). For subsequent analysis purposes we worked with ten degree bins, offset every five degrees.

### The Control Group and Defining the Baseline

Each member of the test-set also has an associated control group. Given the three day observation period, we step through the 44 year archival time period (Fig. 1c) creating a control group of 5,355 observation periods that do not overlap the test event. Using the latitude and longitude of the test event as the local origin, we generate a histogram of the observed counts for each member of the control group. The binned counts of the control group are then added together to define a baseline count of seismic activity as seen from the frame of reference of the given member of the test set under examination. Dividing the total count by the size of the control group gives the expected numbers for a single three-day period (Fig. 1e).

### Filtering out Aftershocks

Care is taken to remove known clustering processes from this analysis using a standard declustering technique. The number of aftershocks follow distribution patterns based on the magnitude of the main event, such as the ‘epidemic type’ model^30^. Our concern here is to remove the additional earthquakes that follow from a main event which would anomalously elevate our counts in the three-day observation window, as well as to decluster the data over time and remove their effects from the baseline statistics.

The declustering method selected was the windowing technique applied by Gardner and Knopoff^31^. While this method is easy to implement, importantly the temporal distribution of the earthquakes in the declustered catalog follows a Poisson distribution^31,32^. Using this method, an earthquake has both a spatial and a temporal extent which are functions of magnitude. The equations used are given in equation (1) of van Stiphout *et al*.^32^. Specifically, the distance window (in kilometers) is estimated by 10^0.1238 M+0.983^. The time window (in days) has one curve for M6.5 and larger (10^0.032 M+2.7389^) and another for smaller magnitudes (10^0.5409M−0.547^). The temporal extent can be quite significant, as data from large magnitude events extend hundreds of days. For example, the time window for M5.0 covers 144 days, M6.0 goes 499 days, M7.0 is at 918 days, and M8.0 extends to 988 days. Similarly, the distance window for M5.0 covers 40 km, M6.0 is at 53 km, M7.0 spans 71 km, and M8.0 extends to 94 km. Data that fall in the combined spatial and temporal windows, either before or after a main event, are flagged as aftershocks or foreshocks and removed from the subsequent analysis.

In an attempt to minimize the impact of aftershock data that may get by the above declustering method from contaminating our three-day test statistics, we will be omitting bins close to the source that may be impacted by these data. The spatial extent of this aftershock zone is set at 3 times the rupture length of each event^18^. Rupture length was estimated from moment magnitude following the 24-hour aftershock distribution linear equations of Kagan and Jackson^33^ (2013) (their Fig. 9). Magnitudes reported at M6.2 or greater were considered equivalent to Mw estimates. For the largest earthquakes this exclusion zone extends out to 25 degrees, and the first ten degree bin for these data is centered at 30 degrees. For M7.5 earthquakes, the exclusion zone extends out to 3.5 degrees. For M7.5 earthquakes (and smaller) we will be omitting the first bin closest to the source, and starting with the bin centered at 10 degrees.

The intent in this step is to minimize the aftershock contamination of the three-day window statistics for our various test sets, and to leave the data in a Poisson distribution (Fig. 1d). If the initial declustering windows applied from Gardner and Knopoff (1974) allow some aftershocks into the baseline statistics, it will increase the baseline statistics slightly and decrease the resulting relative rates accordingly.

### *Experiment 2*: A retrospective analysis testing for source events

*Experiment 2*, looking backward in time, is an alternative design for a numerical experiment to see if large events trigger subsequent earthquakes and allows us to probe the sensitivity of triggering to the size of the triggered event. We start with test-sets of various magnitudes (M5.0 through ≥M6.5) which now represent potentially triggered events. By examining the distribution of large earthquakes (≥M6.5) in the previous three days time, we obtain patterns in potential source events. As in *Experiment 1*, we exclude all earthquakes that are flagged as aftershocks or foreshocks. We also ignore the first 25 degrees of potential aftershocks from the largest sources, and the first ten degree bin for these data is centered at 30 degrees.

### *Experiment 1* vs *Experiment 2*

In *Experiment 1*, the test-set represents possible source events, and only large events are selected (typically, ≥M6.5). The observed counts obtained by looking forward in time represent possible triggered events and all magnitudes are tallied (≥M5.0). The baseline distribution represents the average distribution of earthquakes ≥M5.0. In *Experiment 2*, the test-set represents possible triggered events and all magnitudes are now possible (≥M5.0). The observed counts obtained by looking backward in time represent possible source events and only large magnitudes are tallied (≥M6.5). The baseline distribution now represents the average distribution of earthquakes ≥M6.5. In theory, *Experiment 1* and *Experiment 2* are independent measures of the same global forcing function.

The result of both *Experiment 1* and *Experiment 2* is a single run of a numerical experiment for a given magnitude test-set. In both cases we have observed counts for each of the test ‘subjects’, as well as associated baseline counts from their control groups of 5,355 observation periods. Total observed counts and total baseline counts (as a function of arc-distance) are then provided for the calculation of *p*-values.

### Calculation of *p-*values

The general binomial distribution is of the form

1 $$f(x)=[n!/(n-x)!]\ast {p}^{x}\ast {(1-p)}^{(n-x)}$$

The first part (with factorials) is generally read “*n* choose *x*” where ‘*x*’ is the number of successes out of ‘*n*’ trials, and the probability ‘*p*’ gives the probability of a success.

Each test-set supplies earthquake counts observed over three days with respect to the local origin, *ni*,*j*,*1*, where ‘*i*’ represents the ith event in the test set and *j* = 1, 2, …, J = 180 represents the 1° bins for the observed counts (*Supplemental Information*). The number of observed counts plays the role of ‘*x*’ in the binomial distribution. Each test set also supplies the number of baseline counts observed by the control group, *ni*,*j*,*0*, which, when added to *ni*,*j*,*1*, becomes the total number of events possible (or ‘*n*’ in the binomial ‘*n* choose *x*’ expression). The probability of success, based on the null hypothesis, is the same for all the individual observation periods of the control group (5,355 members) and the single observation period of the test set (i.e., *p* = 1/5,356). It is also the same for all arc-distances, as the odds determination of the relative risk effectively cancels out the baseline variability (Supplemental Information, equation S3). While this description is for 1° bins, the same approach can be extended to the 10° bins shown in this paper.

Putting these together, what is needed is a cumulative probability function for the binomial distribution which will give the total probability (according to the null hypothesis) of getting up to ‘*x*’. In R^34^, this is given by the function ‘pbinom’. If *cpf* = pbinum(*count*, *size*, *prob*), then the *p-*value is given by 1 – *cpf*. The *p-*value thus gives the probability that the null hypothesis could deliver a count of ‘*x*’ or more.

The last detail is that we calculate the mid *p*-value^35^ using [*count* – 0.5] instead of the traditional *p*-value, to avoid the known bias appearing in the binomial calculation based on discrete quantity that take only integer numbers (such as earthquake counts).

### Normalizing relative rates to a common sample size

In both *Experiment 1* and *Experiment 2* we have numerical experiments run with different sample sizes. As sample size increases, the relative rate associated with a given *p*-value drops accordingly. If we wish to compare relative rates between different numerical experiments, it is necessary to adjust the relative rates to a common size, while requiring them to maintain the same *p*-value.

Relative rates are a function of the number of samples (ns) and the number of controls (nc), while the *p*-value is a function of the number of samples (ns) and the total number of trials (ns + nc). If we wish to adjust the number of samples to new values (new_ns), then we must find a new number of controls (new_nc) that yields the closest possible *p*-value as before. Once we have a new_nc to go with the new_ns, we can calculate an adjusted (or normalized) relative rate.

The added detail here is that we scale the individual bin counts proportionally to the amount that ns changes. For example, if we wish to go from a test set of 50 to a test set of 100, all bin counts are doubled, and new_nc values are then calculated to come as close as possible to the previously calculated *p*-value for the given bin.

## Electronic supplementary material

Mathematical framework
