# Supplementary material for: Evidence of Systematic Triggering at Teleseismic Distances Following Large Earthquakes
Source: Sci Rep. 2018 Aug 2;8:11611. doi: 10.1038/s41598-018-30019-2 (PMC6072761; doi:10.1038/s41598-018-30019-2)
Supplement: Supplementary file 1 — Mathematical framework [file 41598_2018_30019_MOESM1_ESM.pdf]

Supplementary Information for:

EVIDENCE OF SYSTEMATIC TRIGGERING AT TELESEISMIC DISTANCES FOLLOWING LARGE EARTHQUAKES

Robert T. O'Malley[1,*], Debashis Mondal[2], Chris Goldfinger[3] & Michael J. Behrenfeld[1]

---

1 Department of Botany of Plant Pathology, Cordley Hall 2082, Oregon State University, Corvallis, OR 97331-2902, USA (email) omalleyr@science.oregonstate.edu (phone: 541-737-2316)

2 Department of Statistics, 239 Weniger Hall, Oregon State University, Corvallis, OR 97331-4606

3 College of Earth, Ocean, and Atmospheric Sciences, 104 CEOAS Administration Building, Oregon State University, Corvallis, OR 97331-5503, USA

April 24, 2018

* To whom correspondence should be addressed

1 Earthquake data and the null probability model

Let $E_1, E_2, \ldots, E_I$ be I large source events (i.e., earthquakes) that occurred respectively at locations $x_1, x_2, \ldots, x_I$ and at time points $t_1, t_2, \ldots, t_I$. Let $n_{i,j,1}$, $j = 1, 2, \ldots, J = 180$, denote the number of moderate to large earthquakes that occurred within $(j - 1, j]$ arc-distance away from x_i in the p days period $(t_i, t_i + p)$ following the occurrence of earthquake E_i. The focus here is on *Experiment 1*. Typically, it is believed that earthquakes occur independently of each other beyond the aftershock region with a rate that may vary according to the local geological and tectonic conditions. In the analysis we therefore remove potential aftershocks as described in the Methods section of the main text. Thus, the law of small numbers[1] applies and we can assume that the counts $n_{i,j,1}$, $i = 1, \ldots, I$, j = 1, 2, … , 180, are independent Poisson random variables with rates (means) $\lambda_{i,j,1}$. To assess whether the risk of occurrence of an earthquake is elevated in a systematic way following the occurrence of a large earthquake E_i, we also consider baseline earthquake counts $n_{i,j,0}$ that occurred within $(j - 1, j]$ arc-distance away from x_i in a period of M x p days excluding the 2p day periods $(t_i - p, t_i + p)$ which overlap with the occurrence of earthquake E_i. For each earthquake E_i, these baseline counts $n_{i,j,0}$ are obtained from all moderate lo large earthquakes that occurred in the time period 1973 to 2016. Since we removed potential aftershocks, the counts $n_{i,j,0}$, $i = 1, \ldots, I$, j = 1, 2, … , 180, are approximately independent Poisson random variables with respective baseline rates (means) $\lambda_{i,j,0}$. Under the null hypothesis that a large earthquake does not change the risk of occurrences of subsequent earthquakes, we must have

$$H_{0,j} : \lambda_{i,j,1} = \lambda_{i,j,0} / M. \qquad \text{(S1)}$$

However, if the occurrence of a large earthquake increases the risk of occurrences of subsequent earthquakes at arc distance (j – 1, j], then it must follow that

$$H_{A,j} : \lambda_{i,j,1} > \lambda_{i,j,0} / M \qquad \text{(S2)}$$

at least for some value of i and j. Let $n_{i,j,+} = n_{i,j,0} + n_{i,j,1}$, $\lambda_{i,j,+} = \lambda_{i,j,0} + \lambda_{i,j,1}$, $n_{i,+,k} = \sum_j n_{i,j,k}$ and $\lambda_{i,+,k} = \sum_j \lambda_{i,j,k}$. For each earthquake E_i, our count data can then be represented by an I x J table $N_i = (n_{i,j,k})$ with margin totals $n_{i,j,+}$ and $n_{i,+,k}$.

$n_{i,1,1}$	$n_{i,2,1}$	. . .	$n_{i,j,1}$	. . .	$n_{i,J,1}$	$n_{i,+,1}$
$n_{i,1,0}$	$n_{i,2,0}$	. . .	$n_{i,j,0}$	. . .	$n_{i,J,0}$	$n_{i,+,0}$
$n_{i,1,+}$	$n_{i,2,+}$	. . .	$n_{i,j,+}$	. . .	$n_{i,J,+}$	$n_{i,+,+}$

Furthermore, the null model state that the relative risk

$$H_{0,j} : \lambda_{i,j,1} / \lambda_{i,j,+} = 1 / (1+M), \ \mathrm{j} = 1, 2, \ldots, \mathrm{J}, \ \mathrm{i} = 1, 2, \ldots, \mathrm{I}, \qquad \text{(S3)}$$

against the alternative

$$H_{A,j} : \lambda_{i,j,1} / \lambda_{i,j,+} > 1 / (1+M), \qquad \text{(S4)}$$

at least for some j = 1, 2, . . . , J and i = 1, 2, . . . , I.

2 Test statistics, *p*-values and assessment of the null model

Under the null model, the exact conditional distribution of $n_{i,j,1}$ given the total $n_{i,j,+}$ is binomial with parameters $n_{i,j,+}$ and 1/(M+1). Furthermore, since the aftershock-removed events occur independently of each other and are rare in space and time, the counts $n_{i,j,1}$, i = 1, . . . , I, j = 1, . . . , J given total $n_{i,j,+}$, i = 1, . . . , I, j = 1, . . . , J are approximately independent. Let $n_{+,j,1} = \sum_i n_{i,j,1}$ be the total number of events that occur within *p* days period and (j – 1, j] arc-distance away from a source event. Furthermore, let $n_{+,j,+}$ be the total number of events that occurred within (j – 1, j] arc-distance away from a source event in the time period 1973-2016. Since the sum of independent binomial random variables with the same probability of failure is a binomial random variable, it follows that $n_{+,j,1}$, j = 1, . . . , J, conditioned on $n_{i,j,+}$, i = 1, . . . , I, j = 1, . . . , J are approximately independent binomial distributions with respective parameters $n_{+,j,+}$ and 1/(M+1). Thus, under the null model, $n_{+,j,1}$, j = 1, . . . , J, conditioned only on $n_{+,j,+}$, j = 1, . . . , J, are approximately independent binomial distributions with respective parameters $n_{+,j,+}$ and 1/(M+1).

We are primarily interested in the collective behavior of large source events. If occurrences of large source events do not increase the risk of occurrences of other events at teleseismic distances, we would expect that the numbers $n_{+,j,1}$, j = 1, . . . , J, are compatible with independent binomial distributions with respective parameters $n_{+,j,+}$ and 1/(M+1). However, if occurrences of large source events increase the risk of occurrences of other events in a systematic way at teleseismic distances, we

would expect counts $n_{+,j,1}$, j = 1, . . . , J, to also be large in a systematic way in comparison to realizations from independent binomial distributions with respective parameters $n_{+,j,+}$ and 1/(M+1).

For discrete test statistics such as the counts $n_{+,j,1}$, j = 1, . . . , J, it is well recognized that traditional *p*-values for a one-sided test are conservative and do not provide exact significance levels[2-4]. For discrete test statistics, we define the randomized *p*-values[3] as

$$p_{j,R} = \mathrm{pr}\ (B_j > n_{+,j,1}) + U_j \times \mathrm{pr}\ (B_j = n_{+,j,1}), \qquad \text{(S5)}$$

where B_j, $j = 1, \ldots, J$ are independent binomial random variables with parameters $n_{+,j,+}$ and 1/(M+1), and U_j, $j = 1, \ldots, J$ are independent and identically distributed uniform random variables in (0,1). Under the null hypothesis, it follows that $p_{j,R}$, $j = 1, \ldots, J$ are also independent and identically distributed uniform random variables in (0,1). However, if occurrences of large source events increase the risk of occurrences of other events in a systematic way beyond the teleseismic distances, we would expect that $p_{j,R}$, $j = 1, \ldots, J$ are systematically small in comparison with uniform random variables in (0,1).

The conditional expectation of the randomized *p*-value is $p_{j,R}$ is known as the mid-*p*-value[4] and is given as

$$p_{j,M} = \mathrm{pr}\ (B_j > n_{+,j,1}) + ½ \times \mathrm{pr}\ (B_j = n_{+,j,1}). \qquad \text{(S6)}$$

When the null model is true the mean mid-*p*-value is 0.5, and this property make them particularly suitable in summarizing an overall assessment.

Supplemental References

1. Kingman, J. F. C. *Poisson Processes*. Oxford Science Publications. Oxford (1993).
2. Berry, G. and Armitage, P. Mid-*P* confidence intervals: a brief review. *The Statistician*, 417-423 (1995).
3. Cox, D. R. and Hinkley, D. V. *Theoretical Statistics*. CRC Press (1979).
4. Lancaster, H. O. Significance tests in discrete distributions. *Journal of the American Statistical Association*, **56,** 223-234 (1961).
